# Mr. Ring, on Inoculation

**Published:** 1805-07-01

**Authors:** John Ring

**Affiliations:** New Street, Hanover Square


### *To the Editors of the Medical and Physical Journal.*

GENTLEMEN,

DR. MOSELEY has lately published a pamphlet, in which he asserts, that a child of Mr. Curling, in George Street, Portland Chapel, vaccinated by me, has since had the small-pox. The fact is, that the child had a disorder attended with a slight eruption, which Mrs. Curling supposed posed to be the small-pox; but having carried the child to Mr. Leighton, of Welbeck Street, and Mr. Draper, of Bulstrode Street, they both declared it to be the chicken-pox. On this account the case was never communicated to me; nor should I have known it now, but from Dr. Moseley's publication.

Dr. Moseley seems not to be very difficult of conviction on such occasions; for having called on Mrs. Curling, and asked her whether her child had the small-pox after the cow-pock, she answered, that he had, according to her opinion, but that she was not certain; to which Dr. Moseley immediately answered, there was not a doubt of it, for many others had the same.

The case alluded to in the third paragraph of Dr. Moseley's pamphlet, p. 129, I have authority from the family to say, is a misrepresentation of the fact; and the doctor must have received wrong information.

From these, and other circumstances, I am inclined to believe, that the cases published in Dr. Moseley's pamphlet are in general ill-founded; and that he has been too ready to believe what agreed with his pre-conceived hypothesis; but I shall, hereafter, express my sentiments on this subject more fully, in another form.

False alarms are daily spread, concerning vaccination; but there are others which deserve attention. The following is the first instance in which I have ever seen a failure in my own practice; and a clear case of the small-pox after the cow-pock.—A child of Mr. Fairbrother, in Exeter Street, Brompton, vaccinated by me about two years ago, has at this time the remains of a pustulous eruption, which appeared to be variolous, and was in some degree confluent; but it turned on the sixth day, and is rapidly disappearing. Many of those eruptions which first appeared, had vanished by the sixth day; and the complaint has proved more favourable than could be expected.

When this child was vaccinated, which was about two years ago, he laboured under the tinea capitis, which, sometimes, occasions an irregularity in the vesicle; but nothing of that kind was observable in the present instance. I am therefore inclined to suppose, that the morbid action already existing in the habit, partly, or entirely, prevented the vaccine vesicle from producing its full effect on the constitution.

Instances of local cow-pock are already recorded; one of them occurred at the Vaccine Pock Institution, another in the practice of Mr. Forbes; this is the first that has fallen under my observation. Such cases are uncommon;

2 and and occur in variolous, as well as in vaccine inoculation. Hence, the late Dr. Heberden remarks, in his Medical Commentaries, that those persons who have no eruption, after variolous inoculation, and only a pustule on the arm, are not secure from future infection.

The real failures, either in vaccine or variolous inoculation, are certainly but few, in comparison of the number supposed to occur; but errors in both kinds of inoculation are undoubtedly frequent. In the last number of your Journal, I communicated the case of a child in Edgware Road, who has had the small-pox after supposed vaccination. The mother of the child informed me, that her grandfather and grandmother were inoculated for the small-pox, and supposed to have had it regularly; yet, they both afterwards died of that disease.

The following case was lately communicated to me by the Rev. Mr. Jenner.—Miss Price, niece to Mr. Whitebrook, wine-cooper, in Greek Street, Soho, was inoculated for the small-pox, when an infant, by the late Dr. Barwis of Devizes, at the time of a general inoculation in that town. She had the disease in a satisfactory manner; and some marks of it are still visible in her face. About two months ago she sickened with the natural small-pox, and had a pretty full eruption. She had the chicken-pox when a child; but independent of this circumstance, it is not probable a mistake should have been committed, with regard to the matter used in inoculation, when the small-pox prevailed in the town where she resided.

I am, &c.

*New Street, Hanover Square.* JOHN RING.

